# Ticking on Pandora’s box: a prospective case-control study into ‘other’ tick-borne diseases

**DOI:** 10.1186/s12879-021-06190-9

**Published:** 2021-05-29

**Authors:** D. Hoornstra, M. G. Harms, S. A. Gauw, A. Wagemakers, T. Azagi, K. Kremer, H. Sprong, C. C. van den Wijngaard, J. W. Hovius

**Affiliations:** 1 Amsterdam UMC, Center for Experimental and Molecular Medicine, Amsterdam Institute of Infection and Immunology, University of Amsterdam, P.O. Box 22660 (1100 DD), Amsterdam, The Netherlands; 2 National Institute for Public Health and the Environment (RIVM), Center of Infectious Disease Control, P.O. Box 1 (3720 BA), Bilthoven, The Netherlands

**Keywords:** Prospective case-control study, Study protocol, *Ixodes ricinus* ticks, Tick-borne pathogens, Tick-borne diseases, Hard tick-borne fever, Fever after tick-bite

## Abstract

**Background:**

Tick-borne pathogens other than *Borrelia burgdorferi* sensu lato – the causative agent of Lyme borreliosis – are common in *Ixodes ricinus* ticks. How often these pathogens cause human disease is unknown. In addition, diagnostic tools to identify such diseases are lacking or reserved to research laboratories. To elucidate their prevalence and disease burden, the study ‘Ticking on Pandora’s Box’ has been initiated, a collaborative effort between Amsterdam University Medical Center and the National Institute for Public Health and the Environment.

**Methods:**

The study investigates how often the tick-borne pathogens *Anaplasma phagocytophilum*, *Babesia* species, *Borrelia miyamotoi*, *Neoehrlichia mikurensis*, spotted fever group *Rickettsia* species and/or tick-borne encephalitis virus cause an acute febrile illness after tick-bite. We aim to determine the impact and severity of these tick-borne diseases in the Netherlands by measuring their prevalence and describing their clinical picture and course of disease.

The study is designed as a prospective case-control study. We aim to include 150 cases – individuals clinically suspected of a tick-borne disease – and 3 matched healthy control groups of 200 persons each. The controls consist respectively of a group of individuals with either a tick-bite without complaints, the general population and of healthy blood donors. During a one-year follow-up we will acquire blood, urine and skin biopsy samples and ticks at baseline, 4 and 12 weeks. Additionally, participants answer modified versions of validated questionnaires to assess self-reported symptoms, among which the SF-36, on a 3 monthly basis.

**Discussion:**

This article describes the background and design of the study protocol of ‘Ticking on Pandora’s Box’. With our study we hope to provide insight into the prevalence, clinical presentation and disease burden of the tick-borne diseases anaplasmosis, babesiosis, *B. miyamotoi* disease, neoehrlichiosis, rickettsiosis and tick-borne encephalitis and to assist in test development as well as provide recommendations for national guidelines.

**Trial registration:**

NL9258 (retrospectively registered at Netherlands Trial Register, trialregister.nl in in February 2021).

**Supplementary Information:**

The online version contains supplementary material available at 10.1186/s12879-021-06190-9.

## Background

Tick-borne diseases (TBDs) have been predicted to increase in Europe due to a combination of human behaviour, climatic and environmental changes [1]. Annually tens of thousands of European patients acquire Lyme borreliosis (LB) and tick-borne encephalitis (TBE) [2–5]. In contrast, the prevalence and impact of other TBDs, in Europe in general and in the Netherlands in particular, are currently unknown [6–11]. These TBDs are caused by several tick-borne pathogens (TBPs) – including *Anaplasma phagocytophilum*, *Babesia divergens*, *Babesia microti*, *Babesia venatorum*, *Borrelia miyamotoi*, *Neoehrlichila mikurensis*, *Rickettsia helvetica* and *Rickettsia monacensis* – which are all detected in *Ixodes ricinus* in the Netherlands [7, 12–19].

With annually approximately one and a half million tick-bites in the Netherlands alone [11] and roughly one third of the ticks being infected with at least one potential TBP other than *Borrelia burgdorferi* sensu lato [19], numerous individuals are exposed to other TBPs each year. Recent molecular detection data indicate that the probability of infection with another TBP after a tick-bite in the Netherlands is 2–3%, which is comparable to the risk of contracting LB [20]. It should be noted that – in analogy to an infection with *B. burgdorferi* s.l. – not everyone who gets infected with another TBP will develop symptomatic disease. Nevertheless there is mounting published and unpublished data showing molecular and serological evidence of infection with various TBPs in humans in Europe, including the Netherlands, in both asymptomatic and symptomatic patients [21–32]. These are the main reasons why the Minister of Health and the National Health Council [33], requested more insight in the public health relevance of other TBPs.

The clinical course of TBDs other than LB varies widely and can range from a typical febrile illness several weeks after a tick-bite [30, 34] to a less frequent severe illness and even death in immunocompromised patients, depending on the TBP [28, 34]. Moreover, co-infections with multiple TBPs appear to be able to alter the course of acute LB [35–41]. However, even in endemic regions in the USA and Europe, co-infections seem to only occur occasionally [21, 24, 42–44].

Lack of awareness, case definitions, laboratory diagnostics, as well as a non-characteristic clinical presentation are among the reasons why these other TBDs often go undiagnosed [19, 45]. With the current study, we aim to determine the impact and severity of other TBDs in the Netherlands by measuring their prevalence and describing their clinical picture and the course of disease.

## Methods/design

### Study design

This is a prospective case-control study with a one-year follow-up to assess the disease prevalence and impact of the TBDs anaplasmosis, babesiosis, *B. miyamotoi* disease (BMD), neoehrlichiosis, rickettsiosis and TBE after a tick-bite in the Netherlands. For this purpose, cases – individuals who develop fever after tick-bite – are compared with several healthy control groups. This study represents a collaborative effort between Amsterdam University Medical Center (former AMC, Amsterdam, the Netherlands) and the National Institute for Public Health and the Environment (RIVM, Bilthoven, the Netherlands). The study has been approved by the Medical Ethics Committee of the Amsterdam UMC (registration no. NL61446.094.17), and is conducted according to the principles of the Declaration of Helsinki.

### Study population

**Fig. 1:**
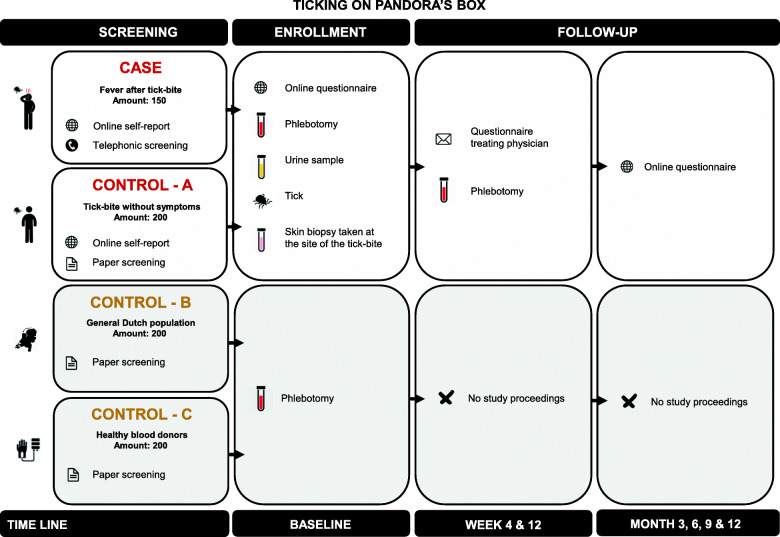
Flowchart of study design

We aim to enroll four groups in the case-control study (Fig. 1). Cases are 150 adults from the Netherlands who report a febrile episode within 4 weeks after tick-bite (Table 1). Strict case descriptions are used for the selection, which includes an objectified fever (measured temperature ≥ 38.0 °C) within the last 4 weeks, developed in the course of 4 weeks after tick-bite. Cases who have not seen the tick attached or who describe signs and symptoms that have another reasonable cause besides a TBD, are excluded. We will also include three type of healthy control participants, consisting of 200 tick-bite controls (A), 200 randomly sampled general Dutch population controls (B) and 200 blood donor controls (C). These groups are frequency matched with the cases by age and gender. Furthermore, both the tick-bite (A) and blood donor controls (C) frequency match to the cases by geographical residence. The tick-bite controls (A) also match by month of tick-bite. All individuals aged ≥16 years reporting on the online inclusion platform or visiting the outpatient department of infectious diseases of the Amsterdam UMC are eligible.

**Table 1:** Inclusion and exclusion criteria

**Cases**	
Inclusion criteria:	
• Subjects are ≥16 years old;	
• Subjects report a tick-bite acquired within the last 2 months;	
• Subjects report an objectified (measured rectally, axillary, orally or tympanic) fever (defined as ≥38.0 °C) within the last 4 weeks, developed in the course of 4 weeks after tick-bite;	
• Subjects live or stay in the Netherlands during the course of the study.	
Exclusion criteria:	
• Subjects with evident signs or symptoms of another cause of the fever besides a TBD;	
• Subjects unable to provide informed consent or do not have sufficient proficiency of the Dutch language.	
**Tick-bite controls (A)**	
Inclusion criteria:	
• Subjects are ≥16 years old;	
• Subjects report a tick-bite acquired within the last 2 months;	
• Subjects frequency match to cases by gender, age, province of residence and month of tick-bite acquirement;	
• Subjects live or stay in the Netherlands during the course of the study.	
Exclusion criteria:	
• Subjects develop an objectified (measured rectally, axillary, orally or tympanic) temperature > 37.3 °C within 4 weeks after the tick-bite;	
• Subjects with evident signs or symptoms of a currant infectious disease;	
• Subjects unable to provide informed consent or do not have sufficient proficiency of the Dutch language.	
**Population background controls (B)**	
Inclusion criteria:	
• Subjects are ≥16 years old;	
• Subjects participating in the national serosurvey PIENTER-3 of the National Institute for Public Health and Environment (RIVM) of the Netherlands;	
• Subjects frequency match to cases by gender and age.	
Exclusion criterium:	
• Subjects unable to give informed consent or do not have sufficient proficiency of the Dutch, English, French, Spanish, Arabian, Turkish or Papiamentu language.	
**Healthy blood donors (C)**	
Inclusion criteria:	
• Subjects are ≥18 years old;	
• Subjects presenting as a healthy blood donor at the national blood bank Sanquin;	
• Subjects frequency match to cases by gender, age and province of residence.	
Exclusion criterium:	
• Subjects unable to give informed consent.	

### Recruitment, inclusion and follow-up of participants

Recruitment started in May 2018. The cases and tick-bite controls (A) are prospectively recruited in a similar manner as described in the study protocols of LymeProspect [46] and VICTORY [47]. The majority of the cases are recruited online, however some participants are additionally included through the outpatient department of infectious diseases of the Amsterdam UMC after evaluation by one of the investigators. The recruitment procedures, and in- and exclusion criteria for the cases are identical in both routes. The tick-bite controls (A) are solely recruited online.

Recruitment, inclusion and follow-up of both the cases and tick-bite controls (A) occur online at www.tekenradar.nl, a secured online platform operated by the RIVM and Wageningen University. People can visit the website after referral by their treating physician or on their own initiative. Cases thus recruited primarily have fever after a tick-bite, whereas the tick-bite controls (A) exclusively report a tick-bite without meeting any criteria of a possible infectious disease diagnosis. Written informed consent is obtained from all eligible participants. Importantly, to verify the complaints or diagnosis after online enrollment the participant’s treating physician is consulted. Blood collection can be done locally or in the participating hospital and is performed at baseline, after 4 weeks and on an optional basis after 12 weeks (Table 2). In addition to the first phlebotomy at baseline, the participants are asked to send in a urine sample and the tick that has bitten them. Cases with skin manifestation have the option of giving additional informed consent for skin biopsy.

**Table 2:** Data collection and measurements for study participants. Cases and tick-bite controls

	Baseline	4 weeks	3 months	6 months	9 months	12 months
Written information and informed consent	X					
Baseline characteristics	X					
Physical examination	X^a^	X^a^				
Recording of clinical manifestation, treatment and concomitant medication	X		X	X	X	X
Recording of adverse events	X		X			
**Laboratory measurements**						
General laboratory measurements	X	X^b^	X^b, c^			
Cultures - TBPs	X^d^					
Multiplex qPCR - TBPs	X	X	X^c^			
C6 EIA and confirmatory immunoblot – *Borrelia burgdorferi* s.l.	X	X	X^c^			
ELISA and confirmatory VNT - TBEV	X	X	X^c^			
IFA – *Anaplasma phagocytophilum, Babesia microti, Rickettsia* SFG	X	X	X^c^			
Immunofluorescence protein microarray – *Borrelia miyamotoi*	X	X	X^c^			
**Questionnaires**						
Comorbidities and pre-existent symptoms	X		X	X	X	X
Somatic symptoms	X		X	X	X	X
Pain, physical and social functioning: SF-36	X		X	X	X	X
Relevant medical information from the treating physician		X				

For explanation of abbreviations, see the main text

^a^Participants included through the outpatient department of infectious diseases of the Amsterdam UMC

^b^General laboratory measurements are repeated only at a consecutive time-point when deviating at baseline

^c^Laboratory measurements are only performed when the third optional phlebotomy is performed

^d^Cultures are performed for participants meeting case criteria only

The two other healthy control groups (B and C), are recruited in a separate way to match the cases. Firstly, the general Dutch population controls (B) are retrospectively selected from the existing national serosurvey PIENTER-3, which was carried out throughout 2016–2017 [48]. Additionally, we prospectively recruited blood donor controls (C) from the National blood bank Sanquin in 2018–2019, for which an independent local protocol was approved. In both study arms, written informed consent was obtained and a singular phlebotomy performed at baseline (Table 3). Details on these two study arms are provided in the supplemental material.

**Table 3:** Data collection and measurements for study participants. General Dutch population and healthy blood donors

	Baseline	4 weeks
Written information and informed consent	X	
Baseline characteristics	X	
Recording of adverse events	X	X
**Laboratory measurements**		
C6 EIA and confirmatory immunoblot – *Borrelia burgdorferi* s.l.	X	
ELISA and confirmatory VNT - TBEV	X	
IFA – *Anaplasma phagocytophilum, Babesia microti, Rickettsia* SFG	X	
Immunofluorescence protein microarray – *Borrelia miyamotoi*	X	

For explanation of abbreviations, see the main text

### Epidemiological and clinical measurements

For all participants in the four study arms standard demographical characteristics are available, including age, gender and place of residence. Both cases and tick-bite control (A) participants also provide information on comorbidities and details on previous tick exposure and previous or current episodes of TBDs at baseline and throughout follow-up. Photographs of skin manifestations after tick-bite are obtained for blinded evaluation by independent experts (Table 2). For the general population control group (B) comorbidities and details on tick exposure and any previous episodes of LB are reported (Table 3).

### Laboratory measurements

At baseline general laboratory measurements are performed in a two-step model on the blood samples to gain insight in the general health status of the participant. Thereafter different study materials are used to provide evidence of a current TBD. For that purpose, specific cultures, molecular assays and serology are performed in an attempt to find evidence of laboratory confirmed LB, anaplasmosis, babesiosis, BMD, neoehrlichiosis, rickettsiosis and/or TBE. Details on the various tests are provided in the supplemental material. All samples are processed blinded, i.e. without any markings related to the identity of the participant, type of participant or time-point.

### Questionnaires

Cases and tick-bite control (A) participants will be asked to fill out online questionnaires in both the screening phase as throughout the follow-up phase. Primarily, some screening questions will be displayed, checking for inclusion criteria. In addition, a study physician will screen for other causes of the fever in the medical history of the participant by telephonic interview. Participants with other probable causes of fever are excluded. During a one-year follow-up participants will fill out 3-monthly questionnaires on determinants of health, assessment of symptoms, co-morbid disorders and pre-existing symptoms. These questionnaires consist of modified versions of validated questionnaires, among which the physical and social functioning and the severity and impact of pain through the SF-36 [49, 50], and are specified in the supplementary material.

### Outcome measures and data analysis

The primary outcome measures the prevalence of the different TBPs tested in blood, urine and skin biopsy in a group of participants who develop fever within 4 weeks after tick-bite in the Netherlands, in whom other causes of the fever are excluded. This can either be through direct laboratory (molecular or culture) or indirect (serology) evidence. This measurement will be compared to the prevalence of infection with the same TBPs in the different control groups.

The secondary outcomes measure the long term sequelae and the clinical manifestations as well as the course of disease of participants with evidence of an infection with the different TBPs. These data will be obtained from the questionnaires and information from the treating physicians and measured by laboratory tests, culture, molecular and serological analyses in both cases and control groups.

### Sample size

Based on our previous research [20] we postulate that in circa 3% (0,03) of the tick-bite controls and in 12% (0,12) of the cases evidence of TBD will be found. Assuming to include 200 tick-bite controls and 150 cases this would yield a power of 89% (alpha < 0,05) to detect a significant difference of disease prevalence between the cases and controls. Additionally, we will include 200 general Dutch population and 200 healthy blood donor controls to enhance this outcome. Details on the power calculations are provided in the supplementary material.

## Discussion

The Pandora study aims to evaluate the prevalence of the different TBDs in cases compared to several control groups, together with the determination of clinical manifestations and long term sequelae of the different TBDs. Additionally, acquired study materials – such as bodily materials from well-defined patients and clinical TBP isolates – will serve to validate, improve and develop diagnostic tests.

In general, not much is known about the prevalence and disease burden of the TBDs *Anaplasma phagocytophilum*, *Babesia divergens*, *Babesia microti*, *Babesia venatorum*, *B. miyamotoi*, *Neoehrlichila mikurensis*, *Rickettsia helvetica*, *Rickettsia monacensis* and tick-borne encephalitis virus (TBEV) in the Netherlands [20, 30]. Nevertheless they have been observed repetitively in a vast amount of eco-epidemiological studies on *Ixodes ricinus* [11, 15, 51, 52]. Altogether, with the increasing risk of exposure to and infection with these TBPs, it is crucial to obtain better insight into the risk of disease development.

The main explanation for the presumed underdiagnosis of these other TBDs, is that current diagnostic tools for the TBDs – with exception of TBE – are virtually non-existent, of questionable quality, or poorly validated in a European setting [19]. As a consequence, the awareness of other TBDs among physicians and the public is low. Therefore, to gain more knowledge on the prevalence and nature of TBDs in the Netherlands it is imperative to improve laboratory diagnostic tests.

Our study is unprecedented compared to other studies on other TBDs in Europe, as the patient populations are strictly defined according to consensus criteria, the study includes both tick-bite and healthy controls from the general population as well as blood donors, and is appropriately powered. Furthermore, a wide variety of supportive laboratory tests will be performed which could aid to provide evidence of disease and thus TBP pathogenicity.

In conclusion, the Pandora study has a translational approach consisting of a prospective case-control study to estimate the prevalence and impact of TBDs in the Netherlands and to acquire materials, which directs further improvement and development of diagnostic tests. The results of this study aims to make substantial contributions to and insights into the clinical manifestation, diagnostics and possible treatment of TBDs in the Netherlands. Such information could be used for the modification of existing national guidelines and could be extrapolated to other Western European countries.

## Supplementary Information

**Additional file 1.** (1) Specifics of laboratory measurements. (2) Questionnaires. (3) Sample size calculation.

## Data Availability

We intend to share our datasets either in a publicly available repository or present it in the main manuscript or additional supporting files in a readable format.

## Abbreviations

ALP: Alkaline phosphatase
ALT: Alanine aminotransferase
AMC: Academic Medical Center
Amsterdam UMC: Amsterdam University Medical Centers
AST: Aspartate aminotransferase
*B. burgdorferi* s.l.: *Borrelia burgdorferi* sensu lato
*B. miyamotoi*: *Borrelia miyamotoi*
BMD: *Borrelia miyamotoi* disease
CCMO: Central Committee on Research Involving Human Subjects
CRP: C-reactive protein
DFM: Dark field microscopy
EDTA: Ethylenediaminetetraacetic acid
EIA: Enzyme immunoassay
ELISA: Enzyme-linked immunosorbent assay
GGT: Gamma-glutamyl transferase
IFA: Immunofluorescence assay
IgG: Immunoglobulin G
IgM: Immunoglobulin M
LB: Lyme borreliosis
LDH: Lacate dehydrogenase
MCV: Mean corpuscular volume
MKP: Modified Kelly-Pettenkofer
MLST: Multilocus sequence typing
PHQ-15: Patient Health Questionnaire
RBC: Red blood cell
RIVM: Dutch National Institute for Public Health and the Environment
(RT-)PCR: (Real-time) polymerase chain reaction
SF-36: SFG: Spotted fever group; Health Status Inventory
TBD: Tick-borne disease
TBE: Tick-borne encephalitis
TBEV: Tick-borne encephalitis virus
TBP: Tick-borne pathogen
TiC-P: Trimbos/iMTA questionnaire for Costs associated with Psychiatric Illness
VNT: Virus neutralization test
WBC: White blood cell
