# Supplementary material for: Ticking on Pandora’s box: a prospective case-control study into ‘other’ tick-borne diseases
Source: BMC Infect Dis. 2021 May 29;21:501. doi: 10.1186/s12879-021-06190-9 (PMC8164744; doi:10.1186/s12879-021-06190-9)
Supplement: Supplementary file 1 — Additional file 1. (1) Specifics of laboratory measurements. (2) Questionnaires. (3) Sample size calculation. [file 12879_2021_6190_MOESM1_ESM.docx]

**Supplementary materials**

1. **Laboratory measurements**

*General laboratory measurements*

At baseline the general laboratory measurements are performed in a two step model. The first step of basic measurements are performed for all cases and tick-bite control (A) participants. When at least one outcome of these measurements exceeds the set cut-off values, an additional set of measurements will be done in the second step. At the consequtive time-points of 4 and 12 weeks, when a convaleascent phlebotomy is performed, the measurements are only reapeated when the baseline results deviate from set cutt-offs.

|  | T=0 |  | T=4 weeks |  | T=12 weeks |
| --- | --- | --- | --- | --- | --- |
|  |  |  |  |  |  |
| Step 1 | Basic measurements | normal | No measurements |  | No measurements |
|  |  |  |  |  |  |
|  | Deviating from set cut-off |  | Repeat basic measurements |  | Repeat basic  measurements |
|  |  |  |  |  |  |
| Step 2 | Additional measurements | normal | No measurements |  | No measurements |
|  |  |  |  |  |  |
|  | Deviating from set cut-off |  | Repeat additional measurements |  | Repeat additional  measurements |

The basic measurements consist of C-reactive protein (CRP) on the heparinized plasma and on the EDTA whole blood the general hematologic measurements will be done, consisting of red blood cell (RBC) count with a mean corpuscular volume (MCV), white blood cell (WBC) count with cell differentiation and a thrombocyte count. The additional measurements performed on both the heparinized plasma and ethylenediaminetetraacetic acid (EDTA) whole blood comprise of reticulocytes, haptoglobin, lacate dehydrogenase (LDH), bilirubin (direct and indirect), alanine aminotransferase (ALT), aspartate aminotransferase (AST), alkaline phosphatase (ALP), Gamma-glutamyl transferase (GGT) and creatinine.

| **Measurement** | **Reference values** | **Cutt-off values resulting in additional measurements** |
| --- | --- | --- |
| CRP | 0 - 5 mg/L | ≥ 20 mg/L |
| RBC | 8,5-10,5 mmol/L | ≤ 7 mmol/L |
| WBC | 4,0-10,5 * 10^9^/L | ≤ 4 * 10^9^/L and ≥ 10,5 * 10^9^/L |
| Thrombocytes | 150-400 * 10^9^/L | ≤ 150 * 10^9^/L |

*Giemsa stained whole blood smear*

From the baseline EDTA whole blood sample an automized Giemsa stained blood smear will be made. This slide will be used for a manual WBC differentiation, when the automized measurement proves insufficient, and to asses the potential presence of the intracellular pathogens *Anaplasmosis phagocytophilum* and *Babesia* species by light field microscopy [1].

*Cultures*

At baseline the heparinized plasma will be pelleted for the case participants to innoculate into the *B. miyamotoi* culture medium, Modified Kelly-Pettenkofer (MKP) supplemented with 10% fetal calve serum [2, 3]. Cultures will be passaged once after 3 weeks of inclubation, and weekly analysed by Dark Field Microscopy (DFM) throughout 8 succesive weeks, after which 1,5-2 mL medium will be stored at -80 °C for the molecular analysis of the presence of *Borrelial* DNA.

*Multiplex RT-PCR*

All blood samples of the cases and tick-bite control (A) participants are analyzed with multiplex real-time PCR, based on various fragments of genes specific for *Anaplasma phagocytophilum*, *Babesia microti*, *Babesia* sensu stricto, *B. burgdorferi* s.l., *B. miyamotoi*, *Neoehrlichia mikurensis*, *Rickettsia helvetica,* *Rickettsia* spotted fever group and TBEV [4]. In cases with cutaneous manifestations at site of the tick-bite at study enrollment, a skin biopsy is collected at baseline - after seperate consent - and assessed by multiplex RT-PCR. Additional samples, when present, which are tested by the same molecular method, are the heparinized buffy coats, the stored cultures and the tick that has bitten the study participant. Extraction of whole nucleic acid of the blood samples are performed from 400 μL of EDTA-plasma (Nucleic Acid Isolation Kit I; Roche) using robot-extraction (MagNA Pure Compact Extraction Robot; Roche, Basel, Switzerland). All additional samples are processed manually (Qiagen DNeasy Blood & Tissue Kit). Typing of the isolates from obtained samples is performed by conventional PCR followed by dideoxy-dye termination sequencing [4].

*Serology – B. burgdorferi s.l.*

For detection of *B. burgdorferi* s.l. specific antibodies, a IgM and IgG C6 ELISA (Immunetics, Boston, MA, USA) is performed on all samples, followed by IgM and IgG immunoblot analysis (Mikrogen GmbH, Neuried, Germany) for confirmation of C6 ELISA positive or borderline results [5].

*ELISA and neutralisation assay - TBEV*

An IgG ELISA (TestLine Clinical Diagnostics, Brno, Czech Republic) for TBEV is conducted for all study participants. When reactive, all time-points of that study participant are successively tested for the presence of specific IgM antibodies. Finally all reactive IgM and/or IgG samples are confirmed through a virus neutralization test (in-hous assay RIVM, Bilthoven, the Netherlands).

*IFA – Anaplasma, Babesia and Rickettsia*

Furthermore, indirect immunofluorescence assays (Focus Diagnostics, Cypress, CA, USA) are used for the detection of antibodies directed at *Anaplasma phagocytophilum*, *Babesia* *microti* and *Rickettsia* SFG. Primary all samples are screened for IgG specific antibodies [1]. When reactive all time-point samples of that participant are subsequently screened for the presence of IgM specific antibodies.

*Protein microarray – B. miyamotoi*

Through an experimental protein micoarray – *B. miyamotoi* specific recominant antigens [6, 7] are printed on a glass slide – specific IgM and IgG seroreactivity is measured.

1. **Questionnaires**

The online questionnaires used in this study consist of part of the questionnaires used in the LymeProspect [8] and VICTORY [9] study, supplemented with specific questions to inquire after physical complaints and explore the possibility of having an infectious disease other than tick-borne. Clinical and cognitive-behavioral parameters are assessed, including somatic symptoms, physical and social functioning (SF-36, subscales physical functioning and social functioning) [10], the severity and impact of pain (SF-36, subscale pain) [11], health care use, co-morbidity and pre-existent symptoms preceding enrolment. In addition the treating physician – most likely the general physician – is asked to fill in a standardized questionnaire to objectify clinical manifestations, diagnosis and/or supply supplementary test results.

1. **Calculations of group sizes**

*Cases and tick-bite control group (A):*

As it is uncertain to which extent the exposure to infected ticks and consequently human infection with either other TBP translates to an actual TBD [12, 13], a power calculation is made by assumptions based on the so far published data. All of the different TBPs may cause a febrile disease several weeks after a tick bite. It is roughly estimated that 3-6% of the tick-bite reports in the Netherlands indicate a subjective fever. With, at the time, 10.000 annual tick-bite reports we predict 300-600 self-reported individuals with fever after a tick-bite. However, with the prediction to select 10% of these reports through both strict inclusion criteria and by accounting for loss to follow-up, we expect to be able to include 30-60 cases a year. Throughout 3 inclusion years this would result in a total of approximately 90-180 cases.

With a nominal 0,05 two-sided significance level the power to detect the difference between the null hypothesis proportion, π0 of 0,12 (cases with TBD) and the alternative proportion, πA, of 0,03 (controls with proof of TBP infection) will be 96-99% when the sample sizes would consist of 300-600 cases and 200 tick-bite controls. Assuming 200 tick-bite controls, even as little as 150 cases will suffice to assess the primary outcome, resulting in a power of 89% to detect the difference between the null hypothesis proportion, π0 of 0,12 (cases with TBD) and the alternative proportion, πA, of 0,03 (controls with proof of TBP infection).

*Other control groups*

No sample size calculation was made for the additional control groups background population (B) and healthy blood donors (C). We aim to include a total of 200 background population controls and 200 healthy blood donors in the observational cohort, as this is similar to the expected number of tick-bite controls.

**References**

1. Wormser GP, Dattwyler RJ, Shapiro ED, Halperin JJ, Steere AC, Klempner MS, Krause PJ, Bakken JS, Strle F, Stanek G *et al*: **The clinical assessment, treatment, and prevention of lyme disease, human granulocytic anaplasmosis, and babesiosis: clinical practice guidelines by the Infectious Diseases Society of America**. *Clin Infect Dis* 2006, **43**(9):1089-1134.

2. Wagemakers A, Oei A, Fikrig MM, Miellet WR, Hovius JW: **The relapsing fever spirochete Borrelia miyamotoi is cultivable in a modified Kelly-Pettenkofer medium, and is resistant to human complement**. *Parasit Vectors* 2014, **7**:418.

3. Koetsveld J, Kolyasnikova NM, Wagemakers A, Toporkova MG, Sarksyan DS, Oei A, Platonov AE, Hovius JW: **Development and optimization of an in vitro cultivation protocol allows for isolation of Borrelia miyamotoi from patients with hard tick-borne relapsing fever**. *Clin Microbiol Infect* 2017, **23**(7):480-484.

4. Jahfari S, Hofhuis A, Fonville M, van der Giessen J, van Pelt W, Sprong H: **Molecular Detection of Tick-Borne Pathogens in Humans with Tick Bites and Erythema Migrans, in the Netherlands**. *PLoS Negl Trop Dis* 2016, **10**(10):e0005042.

5. Healthcare CIfQo: **Dutch guideline for Lyme disease**. *Available on* [*https://wwwrivmnl/sites/default/files/2018-11/CBO%20richtlijn%20Lymeziekte%20definitief%20juli%202013pdf*](https://wwwrivmnl/sites/default/files/2018-11/CBO%20richtlijn%20Lymeziekte%20definitief%20juli%202013pdf) 2013.

6. Wagemakers A, Koetsveld J, Narasimhan S, Wickel M, Deponte K, Bleijlevens B, Jahfari S, Sprong H, Karan LS, Sarksyan DS *et al*: **Variable Major Proteins as Targets for Specific Antibodies against Borrelia miyamotoi**. *J Immunol* 2016, **196**(10):4185-4195.

7. Koetsveld J, Kolyasnikova NM, Wagemakers A, Stukolova OA, Hoornstra D, Sarksyan DS, Toporkova MG, Henningsson AJ, Hvidsten D, Ang W *et al*: **Serodiagnosis of Borrelia miyamotoi disease by measuring antibodies against GlpQ and variable major proteins**. *Clin Microbiol Infect* 2018, **24**(12):1338 e1331-1338 e1337.

8. Vrijmoeth HD, Ursinus J, Harms MG, Zomer TP, Gauw SA, Tulen AD, Kremer K, Sprong H, Knoop H, Vermeeren YM *et al*: **Prevalence and determinants of persistent symptoms after treatment for Lyme borreliosis: study protocol for an observational, prospective cohort study (LymeProspect)**. *BMC Infect Dis* 2019, **19**(1):324.

9. van de Schoor FR, Baarsma ME, Gauw SA, Joosten LAB, Kullberg BJ, van den Wijngaard CC, Hovius JW: **Validation of cellular tests for Lyme borreliosis (VICTORY) study**. *BMC Infect Dis* 2019, **19**(1):732.

10. Stewart JW, Quitkin FM, McGrath PJ, Rabkin JG, Markowitz JS, Tricamo E, Klein DF: **Social functioning in chronic depression: effect of 6 weeks of antidepressant treatment**. *Psychiatry Res* 1988, **25**(2):213-222.

11. Aaronson N, Muller M, Cohen P, Essink-Bot M, Fekkes M, Sanderman R, Sprangers M, te Velde A, E V: **Translation, validation, and norming of the Dutch language version of the SF-36 Health Survey in community and chronic disease populations.** *J Clin Epidemiol* 1998, **51**(11):1055-1068.

12. Sprong H, Azagi T, Hoornstra D, Nijhof AM, Knorr S, Baarsma ME, Hovius JW: **Control of Lyme borreliosis and other Ixodes ricinus-borne diseases**. *Parasit Vectors* 2018, **11**(1):145.

13. Azagi TH, D.; Kremer, K.; Hovius, J.W.; Sprong, H.: **Evaluation of Disease Causality of Rare Ixodes ricinus‐Borne Infections in Europe**. *Pathogens* 2020, **9**(150):1-24.
